# Gamma Oscillations in Human Primary Somatosensory Cortex Reflect Pain Perception 

**DOI:** 10.1371/journal.pbio.0050133

**Published:** 2007-04-24

**Authors:** Joachim Gross, Alfons Schnitzler, Lars Timmermann, Markus Ploner

**Affiliations:** 1 Department of Neurology, Heinrich-Heine-University, Düsseldorf, Germany; 2 Centre for Cognitive Neuroimaging, Department of Psychology, University of Glasgow, United Kingdom; 3 Wolfson Centre for Clinical and Cognitive Neuroscience, School of Psychology, University of Wales, Bangor, United Kingdom; 4 Department of Neurology, Technical University Munich, Munich, Germany; F. C. Donders Centre for Cognitive Neuroimaging, The Netherlands

## Abstract

Successful behavior requires selection and preferred processing of relevant sensory information. The cortical representation of relevant sensory information has been related to neuronal oscillations in the gamma frequency band. Pain is of invariably high behavioral relevance and, thus, nociceptive stimuli receive preferred processing. Here, by using magnetoencephalography, we show that selective nociceptive stimuli induce gamma oscillations between 60 and 95 Hz in primary somatosensory cortex. Amplitudes of pain-induced gamma oscillations vary with objective stimulus intensity and subjective pain intensity. However, around pain threshold, perceived stimuli yielded stronger gamma oscillations than unperceived stimuli of equal stimulus intensity. These results show that pain induces gamma oscillations in primary somatosensory cortex that are particularly related to the subjective perception of pain. Our findings support the hypothesis that gamma oscillations are related to the internal representation of behaviorally relevant stimuli that should receive preferred processing.

## Author Summary

Pain is a highly subjective sensation of inherent behavioral importance and is therefore expected to receive enhanced processing in relevant brain regions. We show that painful stimuli induce high-frequency oscillations in the electrical activity of the human primary somatosensory cortex. Amplitudes of these pain-induced gamma oscillations were more closely related to the subjective perception of pain than to the objective stimulus attributes. They correlated with participants' ratings of pain and were stronger for laser stimuli that caused pain, compared with the same stimuli when no pain was perceived. These findings indicate that gamma oscillations may represent an important mechanism for processing behaviorally relevant sensory information.

## Abstract 3

Magnetoencephalography reveals that gamma oscillations in the somatosensory cortex correlate with the subjective rating of pain and are stronger for laser stimuli that cause pain, compared with the same stimuli when no pain is perceived.

## Introduction

Within the continuous flow of sensory information, a huge number of events compete for neural representation and perception. This sensory overflow requires the selection and preferential processing of relevant information in order to optimize the utilization of cerebral processing resources. Recently, induced neuronal oscillations in the gamma frequency range (about 40–100 Hz) have been suggested to represent one mechanism of the selection and preferred processing of sensory information [1–7]. These induced gamma oscillations represent event-related modulations of neuronal oscillations, are often observed in early sensory cortices and differ from evoked neuronal responses in a lack of phase locking to the sensory stimulus. Functionally, the association between induced gamma oscillations, and selection and preferred processing of sensory stimuli suggests that these responses may not only be related to the physical stimulus attributes, but also related particularly to the subjectively weighted percept of a sensory event.

Painful stimuli signal threats and are therefore of utmost behavioral relevance [8,9]. Thus, we hypothesized that painful stimuli induce gamma oscillations in somatosensory cortices. Moreover, we speculated that these pain-induced gamma oscillations may not only relate to the objective attributes of painful stimuli, but may also particularly reflect the subjective experience of pain. To address this issue, we used magnetoencephalography to record neural responses to noxious stimuli in healthy human subjects. We investigated the effects of noxious stimuli on neuronal activity in the gamma band and related these effects to objective stimulus intensity and subjectively perceived pain intensity. Our results show that pain induces gamma oscillations in the contralateral primary somatosensory cortex. Amplitudes of pain-induced gamma oscillations increase with objective stimulus intensity and subjective pain intensity. However, around pain threshold, perceived stimuli induce significantly stronger gamma oscillations than unperceived stimuli of equal stimulus intensity. These observations provide direct evidence for a close association between induced gamma oscillations and the conscious and subjective perception of behaviorally relevant sensory events.

## Results

First, we aimed to identify and to characterize spatially and temporally pain-induced gamma oscillations in human somatosensory cortices. In 12 healthy male participants, 40 moderately painful cutaneous laser stimuli (intensity 600 mJ) were applied to the dorsum of the right hand. Participants were instructed to passively perceive the stimuli without any further task. The contralateral primary (S1) and bilateral secondary (S2) somatosensory cortices were localized by analyzing the well-known [10] pain-evoked (phase-locked) responses from these areas (Figure 1A). Next, we investigated possible pain-induced gamma oscillations in these areas. To this end, time-frequency representations (TFRs) were calculated for each trial and area. The analysis revealed that pain induces strong and significant increases in gamma power at frequencies between 60 and 95 Hz in the contralateral S1 cortex (Figure 1B). These pain-induced gamma oscillations were observed between 100 ms and 300 ms after stimulus application coinciding with the pain-evoked response from S1 (Figure 1C). Please note that these pain-induced gamma oscillations were observed without any particular task and, thus, do not depend on the task relevance of painful stimuli, but rather on their sensory quality and their inherent behavioral relevance. No pain-induced changes in gamma power were observed in the bilateral S2 cortices. Analysis of amplitude and phase dynamics confirmed that gamma oscillations were not phase locked to stimuli, and therefore represent induced, but not evoked oscillations (Figure 2).

**Figure 1:**
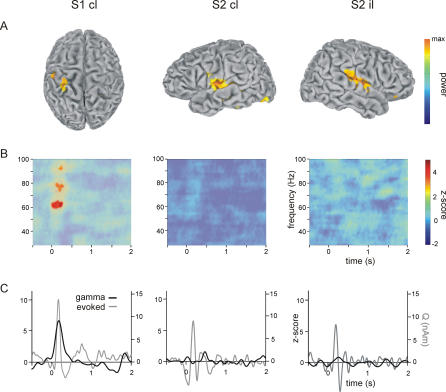
Pain-Induced Gamma Oscillations in Somatosensory Cortices (A) Group mean locations of contralateral primary (S1 cl) and bilateral secondary somatosensory (S2 cl and S2 il) cortices. Locations were obtained from analysis of evoked responses to noxious laser stimuli. Individual tomographic maps of pain-evoked power increases were calculated and averaged across subjects, resulting in a group-mean tomographic map of pain-evoked power increases with dimensionless values (see Methods for details). Talairach coordinates of activations were: −20,−37, and 57 (S1 cl), −45,−15, and 22 (S2 cl), and 50,−16, and 19 (S2 il). The additional colored voxels were not consistently found in single participants and have not been included in further analysis. (B) Group-mean TFRs for each of the three areas. The TFRs show power as a function of time and frequency. Power is coded as *z*-score calculated from a 1,000-ms baseline period. Significance of activations was determined by using permutation statistics; areas below the 95% confidence level are masked by transparent gray shading. Significant oscillations following noxious stimuli (stimulus onset at 0 ms) are evident in contralateral S1 in the high gamma range at a latency of about 200 ms. Please note that the different frequency peaks do not represent harmonics, but result from interindividual variability in frequency of gamma oscillations. No significant oscillations can be seen for bilateral S2 at any time. (C) Group-mean amplitudes of induced gamma oscillations (60–95 Hz, black lines) and evoked activity (gray lines) from contralateral S1 and bilateral S2. The left and right axes and labels correspond to evoked activity and induced gamma oscillations, respectively. Evoked activity is given in source strength and induced gamma oscillations are given in *z*-scores. Evoked activity and induced gamma oscillations in S1 show the same peak latency (evoked: 190 ± 10 ms; induced gamma: 192 ± 15 ms; mean ± the standard error of the mean [s.e.m]; *p* > 0.8, two-tailed Wilcoxon signed-rank test).

**Figure 2:**
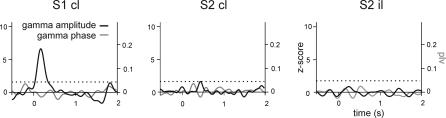
Gamma Amplitude and Gamma Phase Locking in Somatosensory Cortices Gamma amplitude (black line) and gamma phase locking (plv, gray line) are shown for the contralateral primary somatosensory cortex (S1 cl) and the bilateral secondary somatosensory cortices (S2 cl and S2 il). The left and right axes and labels correspond to amplitude and phase, respectively. Amplitudes are given in *z*-scores, and phase is given as phase-locking value (plv; see Methods). The dotted line represents the confidence level as determined from permutation statistics. The increase of gamma oscillations (black line) without a significant change of phase locking (gray line) confirms that gamma oscillations are induced and not evoked.

Second, we investigated the relationship between amplitudes of induced gamma oscillations in S1, stimulus intensity, and perceived pain intensity. To this end, randomly varied intensities of noxious laser stimuli were applied to the right hand of 13 healthy human participants. Possible stimulus intensities were 150, 300, 450, and 600 mJ, which yield sensations ranging from barely detectable to moderately painful. Forty stimuli were presented for each stimulus intensity, and subjects were asked to rate the perception of each stimulus on a scale from 0 to 100 anchored at “no pain” and “worst imaginable pain.” Pain ratings were cued by an auditory signal presented 3 s after each stimulus. The contralateral S1 cortex was localized, and evoked responses and induced gamma oscillations to stimuli of different intensities were analyzed. Mean amplitudes of evoked responses and induced gamma oscillations from S1 were calculated during the time window from 100 ms to 300 ms as compared to baseline amplitudes. Figure 3 shows amplitudes of evoked (phase-locked) responses and induced (non–phase-locked) gamma oscillations as a function of stimulus intensity. The results reveal that amplitudes of induced gamma oscillations and amplitudes of evoked responses from S1 increase with stimulus intensity. This increase in response amplitudes was paralleled by an increase in perceived pain intensity. These observations show that amplitudes of pain-induced gamma oscillations in S1 vary with objective stimulus intensity and subjective pain intensity.

**Figure 3:**
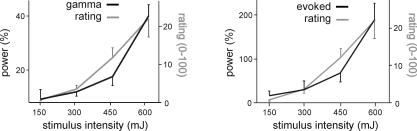
Pain Intensity, Amplitudes of Induced Gamma Oscillations, and Amplitudes of Evoked Responses as a Function of Stimulus Intensity Mean power changes of induced gamma oscillations (black line, left panel) and evoked activity (black line, right panel) at 100–300 ms with respect to baseline were computed for all four stimulus intensities and compared to mean pain ratings (gray lines). Error bars depict ± the standard error of the mean (s.e.m.) Induced gamma oscillations, evoked responses, and pain intensity increase with stimulus intensity. Spearman's correlation coefficient between induced gamma oscillations and pain intensity was 0.96 (*p* = 0.003), and between evoked responses and pain intensity was 0.99 (*p* = 0.012).

Third, we aimed at further defining the relationship between pain-induced gamma oscillations in S1 and the subjective experience of pain. Low intensity (150 and 300 mJ) trials around pain threshold were chosen for the analysis. Per definition, some of these trials are perceived as painful (“percept” trials, rating > 0) and some are not (“no percept” trials, rating = 0). We matched both trials for stimulus intensity and number of stimuli, i.e., for each individual, the “percept” and “no percept” sets of trials did not differ with respect to objective stimulus intensity or number of stimuli, but only with respect to subjective perception. Mean amplitudes of gamma oscillations at 100 ms to 300 ms as compared to baseline were computed for “percept” and “no percept” trials. The analysis reveals that gamma oscillations were significantly stronger for “percept” than for “no percept” trials (*p* = 0.013, Wilcoxon signed-rank test, Figure 4). Gamma power during baseline did not differ between conditions (*p* = 0.15) and only “percept” trials (*p* = 0.003), not “no percept” trials (*p* = 0.735), yielded significant increases of gamma power as compared to baseline. In contrast, amplitudes of phase-locked, evoked S1 responses did not differ between “percept” and “no percept” trials (*p* = 0.38). These observations show that pain-induced gamma oscillations are particularly related to the subjective perception of pain.

**Figure 4:**
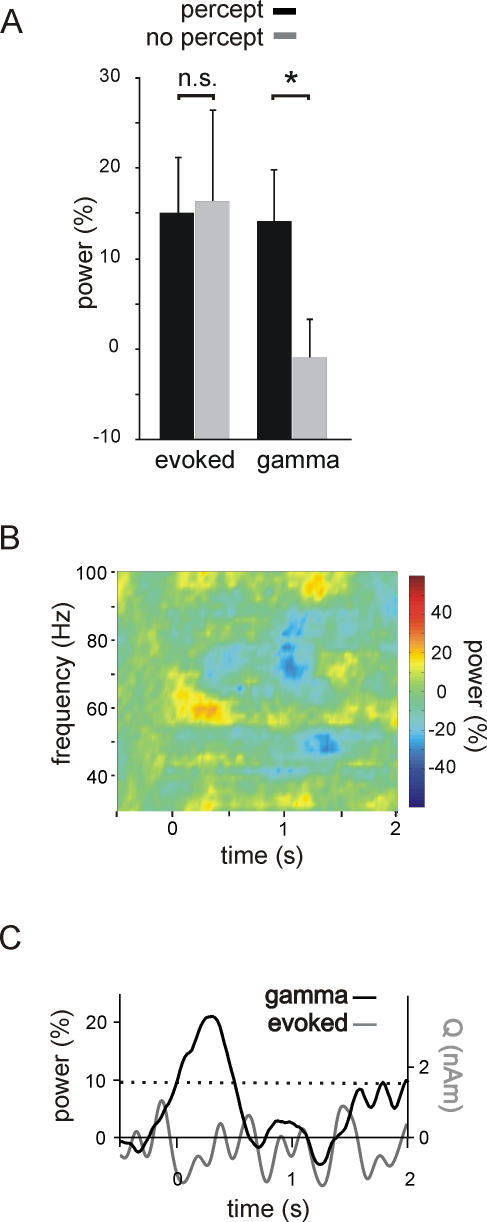
Amplitudes of Induced Gamma Oscillations and Evoked Responses to Differently Perceived Stimuli of Equal Stimulus Intensity (A) Trials rated with zero (“no percept,” black bars) were compared to trials with higher ratings (“percept,” gray bars) but the same stimulus intensity. Amplitudes of responses were calculated as relative power changes as compared to baseline. Mean rating of “percept” trials was seven, the mean number of trials per subject was 16. “Percept” and “no percept” trials were equally distributed across the recording session (ratio of the number of “percept” and “no percept” trials compared across quarters of the recording session; *p* = 0.14; Friedman's analysis of variance). Mean amplitudes of gamma oscillations in S1 at 100–300 ms were significantly stronger for “percept” trials as compared to “no percept” trials. Amplitudes of evoked responses from S1 did not differ between conditions. The asterisk (*) indicates *p* < 0.05. (B) TFR of the difference between “percept” and “no percept” trials. Power is coded as relative power change as compared to baseline. The figure represents a subtraction of the “percept” and “no-percept” TFRs, and demonstrates “percept”-specific enhanced gamma oscillations at a maximum latency of about 200 ms. (C) Group-mean amplitude differences between “percept” and “no percept” trials. The black line shows amplitudes of induced gamma oscillations as relative power changes as compared to baseline, and the gray line shows amplitudes of evoked responses calculated as source strengths from S1. The dotted line represents the 95% confidence interval calculated from baseline. Gamma amplitudes are significantly different between “percept” and “no percept” trials, whereas amplitudes of evoked responses did not differ between trial sets.

## Discussion

Our study demonstrates that painful stimuli induce gamma oscillations in the contralateral S1 cortex. These non–phase-locked gamma oscillations differ from evoked responses in a trial-by-trial jitter in latency, and occur at latencies around 200 ms and at frequencies between 60 and 95 Hz. Amplitudes of pain-induced gamma oscillations increase with both objective stimulus intensity and subjective pain intensity. However, around pain threshold, differences in the subjective perception of objectively similar stimuli were related to differences in amplitudes of induced gamma oscillations. These results show that pain-induced gamma oscillations in S1 are particularly related to the subjective perception of pain.

Here, latencies of pain-induced gamma oscillations between 100 ms and 300 ms indicate that these responses are mediated by A-delta-fibers relating to first pain sensation. Later sensations of warmth or second pain mediated by slowly conducting C-fibers occur at latencies of about 1,000 ms [11], and are unlikely to relate to cortical responses at latencies around 200 ms. Correspondingly, in the present study, “percept” and “no percept” refer to the presence and absence of A-delta-fiber–mediated first pain sensation. Moreover, our findings provide evidence for first pain–related gamma oscillations in S1, but do not preclude gamma oscillations from outside the somatosensory cortices, which were beyond the scope of our analysis.

Induced gamma oscillations have been demonstrated in tasks that require activation and further processing of object representations [12,13]. As common features across sensory modalities, these induced gamma oscillations share a similar timing (around 200 ms to 400 ms) and a focal localization [12,14–16] as compared to low-frequency components. A few previous studies investigated pain-related changes in gamma oscillations [17–19]. However, these studies did not apply selective nociceptive stimuli and could not provide consistent results on location, timing, and functional characteristics of possible pain-induced gamma responses. Other studies investigating the relationship between S1 and S2 responses and pain intensity [20–25] revealed a positive correlation between response amplitudes and pain intensity. However, these studies did not investigate induced but evoked responses or blood oxygen level-dependent (BOLD) effects and, thus, cannot be directly compared to the present results.

Painful stimuli possess utmost behavioral relevance that invariably interrupts ongoing processes, demand full access to system resources, and thereby lead to preferred processing [8,9]. Our study demonstrates that induced gamma oscillations are particularly related to the subjectively weighted percept of noxious stimuli. Thus, our results provide an important link between gamma oscillations and a neural filtering mechanism selecting behaviorally relevant information for action. Indeed, neural oscillations as regular excitability changes of neuronal populations have the capability of gating information flow and adding relevance to spike trains [4,26–28]. Thus, the gamma oscillations observed in the present study may modulate the cerebral processing of painful stimuli and the perceptual quality of the stimulus rather than representing the neural substrate of perception per se. Pain-induced gamma oscillations may thereby participate in activating the sensory representation of a painful stimulus and its selection for further processing. Preferred processing of these stimuli may facilitate behavioral responses aimed at preserving the integrity of the individual.

Beyond, our findings suggest that pain-induced gamma oscillations in S1 are related to a complex cerebral network subserving conscious perception of sensory events. This network includes sensory areas as well as higher order frontal and parietal association cortices [1,29–34]. Within this network, perception appears to depend on the complex relationship between ongoing neuronal activity [30,35], phase-locked evoked responses [30,34], and non-phase-locked–induced [1,32] responses.

Taken together, the present findings show that noxious stimuli induce gamma oscillations in S1 that are particularly related to the subjective experience of pain. These observations are compatible with the hypothesis that induced gamma oscillations are related to the internal representation of behaviorally relevant stimuli which should receive preferred processing. Our findings may, thus, contribute to our understanding of the neural mechanisms of perception and, in particular, to the understanding of the highly subjective experience of pain in health and disease.

## Materials and Methods

### Recordings for experiment 1.

Twelve healthy male participants (mean age: 33 y, range 22–41 y) participated in the experiment. All participants gave informed consent, and the study was performed according to the Declaration of Helsinki with the local ethics committee's approval.

Subjects were comfortably seated in a reclining chair. Forty noxious cutaneous laser stimuli were delivered to the dorsum of the right hand, and subjects were instructed to passively perceive the stimuli with closed eyes [36]. Stimuli were cutaneous laser stimuli that selectively activate nociceptive afferents without concomitant activation of tactile afferents. The laser device was a Tm:YAG-laser (Carl Baasel Lasertechnik, Starnberg, Germany) with a wavelength of 2,000 nm, a pulse duration of 1 ms, and a spot diameter of 6 mm. An optical fiber transmitted the laser beam into the magnetically shielded recording room. Stimulation site was slightly changed after each stimulus. Interstimulus intervals were randomly varied between 10 and 14 s. Applied stimulus intensity was 600 mJ, which evoked moderately painful sensations.

Neural activity was recorded with a Neuromag-122 whole-head neuromagnetometer [37] with passbands of 0.03–170 Hz and digitized with 514 Hz. The exact position of the head with respect to the sensor array was determined by measuring magnetic signals from four coils placed on the scalp. High-resolution T1-weighted magnetic resonance images (MRI) were obtained for each subject. Anatomical landmarks (nasion and preauricular points) were localized in each individual and used for the alignment of the MRI- and magnetoencephalography (MEG) coordinate systems.

### Recordings for experiment 2.

Thirteen healthy, right-handed men participated (mean age: 28 y, range 25–33 y) in experiment 2. In this experiment, stimulus strength varied randomly with possible values of 150, 300, 450, or 600 mJ. Forty stimuli for each intensity were applied. Three seconds after each laser stimulus, an auditory signal prompted the participants to rate the intensity of the initial “pinprick”-like first pain on a rating scale from 0–100. Zero was defined as “no pain,” and 100 was defined as the “worst imaginable pain.” Because the rating was explicitly focused on first pain, a rating of zero did not preclude later sensations of warmth or second pain. The sample rate was 483 Hz, and signals were band-pass filtered between 0.03 and 160 Hz [24]. The other parameters were the same as in experiment 1.

### Analysis.

Recorded signals were high-pass filtered (1 Hz) and visually inspected for artifacts. Contaminated epochs were excluded, leaving a minimum of 36 trials per participants and stimulus intensity.

First, somatosensory cortices were localized by analyzing the well-known pain-evoked responses from these areas [10]. Somatosensory cortices were selected for the analysis because previous studies showed that induced gamma oscillations mainly occur in early sensory cortices [2,7,12,14,16]. To this end, covariance matrices across all sensors were calculated for a prestimulus baseline interval (−400 to 0 ms) and a poststimulus interval (0 to 400 ms) including strongest pain-evoked activity as evident in global field power. From these covariance matrices, neural activity during both intervals was localized by using a spatial filtering algorithm [38–40]. The spatial filter was used with a realistic head model to estimate power in the whole brain, resulting in individual tomographic power maps with voxel sizes of 6 × 6 × 6 mm. For each voxel, the ratio of poststimulus power to prestimulus power was computed resulting in individual functional tomographic power maps that show cortical areas with a strong increase of neural activity following noxious stimuli. These functional maps were individually normalized to one, spatially normalized using SPM2 (Wellcome Department of Cognitive Neurology, Institute of Neurology, London, United Kingdom: http://www.fil.ion.ucl.ac.uk/spm), thresholded at 0.8 of the individual maximum, and averaged across participants. This procedure yields group-mean tomographic maps of pain-evoked power increases with a dimensionless maximum value of approximately 0.4. An arbitrary threshold was used for visualization because only the local maxima, but not the extent of activations, were used for subsequent analysis. The Analysis of Functional Neuroimages/AFNI Surface Mapper (AFNI/SUMA) programs were used for surface rendering (National Institute of Mental Health, Bethesda, Maryland, United States: http://afni.nimh.nih.gov/afni). As in previous MEG studies [10], strongest pain-evoked activity was seen in contralateral S1 and bilateral S2. Note that this analysis aimed at localizing somatosensory cortices and does not preclude activations of additional areas, e.g., insular or cingulate cortex, which may yield lower signal-to-noise ratios due to their deep location and/or radial orientation barely detected by MEG.

Second, individual locations of evoked S1 and S2 responses were optimized beyond the 6-mm grid of the first analysis step. To this end, a multi-dimensional, constrained nonlinear minimization (Nelder-Mead, modified fminsearch function in Matlab, Mathworks: http://www.mathworks.com) was employed. The S1 and S2 maxima from the individual tomographic power maps were used as starting points for the optimization. The position was allowed to change by 1 cm in each direction, whereas orientation was constrained to be tangential to the center of the head. For each position, the ratio of poststimulus activity (0 to 400 ms) to prestimulus activity (−400 to 0 ms) was calculated, and the position with the maximum stimulus-evoked power increase was chosen for further analysis. The same optimization procedure was applied to induced gamma power in S1, revealing that optimized locations of evoked S1 responses and induced gamma oscillations in S1 did not differ (*x*-coordinates, *p* = 0.15; *y*-coordinates, *p* = 0.10; *z*-coordinates, *p* = 0.84; two-tailed Wilcoxon signed-rank tests).

Third, for optimized locations of S1 and S2, time courses of activity were computed for all single trials, using the adaptive spatial filter [38,39]. Note that time courses of all activations were analyzed in source space. These time courses were subjected to a time-frequency analysis based on multi-tapers [41] using the fieldtrip toolbox (F. C. Donders Centre for Cognitive Neuroimaging: http://www.ru.nl/fcdonders/fieldtrip). A multi-taper–based analysis was chosen since this approach provides a robust and optimal way to smooth spectra in the frequency domain, and thereby enhances higher frequency oscillatory components with large-frequency jitter-like induced gamma oscillations. The analysis yields TFRs showing power as a function of time and frequency. TFRs were computed from 30 to 100 Hz in 400-ms–long windows with a spacing of 20 ms between windows. A 400-ms time window was chosen to allow a multi-taper frequency smoothing of ±5 Hz. For each frequency, relative change to a 1,000-ms baseline was computed. Power was coded as *z*-scores calculated from the 1,000-ms baseline. Significance of differences between poststimulus and prestimulus activity in TFRs was determined by applying permutation statistics. To this end, the 12 prestimulus baseline (−1,000 to 0 ms) and the 12 poststimulus (0 to 1,000 ms) parts of the TFRs of the 12 different subjects were randomly permuted 5,000 times. Each time, the maximum difference was computed across time and frequency. The 95th percentile of all 5,000 maximum differences was taken as threshold for the TFRs. This maximum statistics takes multiple comparisons into account [42].

Fourth, in order to distinguish between phase-locked (evoked) and non–phase-locked (induced) neural responses, phase locking of stimulus-related neural activity was determined. For each cortical area, single trial time courses were bandpass filtered (forward and reverse with a fourth-order Butterworth filter) in the gamma frequency band (60–95 Hz) defined from TFRs. The Hilbert transformation yielded instantaneous phase and amplitude estimates for each single trial with an optimum temporal resolution. Phase-locking value (plv, bounded between zero and one) was computed as the absolute value of the mean of complex phase Φ across N trials.

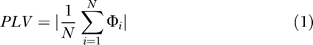
Evoked components show a consistent phase relationship to stimulus that is evident in a high plv. Amplitude and phase dynamics were calculated with reference to a 1,000-ms prestimulus baseline. To establish a confidence level for stimulus-induced phase locking, the time course for each region of interest was randomly permuted 5,000 times and then subjected to the same phase-locking analysis (i.e., filtering, Hilbert transformation, averaging, and baseline correction). The maximum value was used as the confidence level.

## Abbreviations

plv: phase-locking value
TFR: time-frequency representation
